# An Explanation Interface for Healthy Food Recommendations in a Real-Life Workplace Deployment: User-Centered Design Study

**DOI:** 10.2196/51271

**Published:** 2025-02-11

**Authors:** Robin De Croon, Daniela Segovia-Lizano, Paul Finglas, Vero Vanden Abeele, Katrien Verbert

**Affiliations:** 1 Department of Computer Science, KU Leuven, Celestijnenlaan 200A, Leuven, 3001, Belgium, 32 16 37 39 76; 2 Food & Nutrition National Biosciences Research Infrastructure, Quadram Bioscience Institute, Norwich, United Kingdom

**Keywords:** food recommender systems, personalized nutrition, healthy eating, human-computer interaction, real-life deployment, food catering, meal recommendations, nutritional profile, transparency

## Abstract

**Background:**

Despite widespread awareness of healthy eating principles, many individuals struggle to translate this knowledge into consistent, sustainable dietary change. Food recommender systems, increasingly used in various settings, offer the potential for personalized guidance and behavior change support. However, traditional approaches may prioritize user preferences or popularity metrics without sufficiently considering long-term nutritional goals. This can inadvertently reinforce unhealthy eating patterns. Emerging research suggests that incorporating explanations into recommender systems can increase transparency, promote informed decision-making, and potentially influence food choices. Yet, the effectiveness of explanations in promoting healthy choices within complex, real-world food environments remain largely unexplored.

**Objective:**

This study aims to investigate the design, implementation, and preliminary evaluation of a food recommender system that integrates explanations in a real-world food catering application. We seek to understand how such a system can promote healthy choices while addressing the inherent tensions between user control, meal variety, and the need for nutritionally sound recommendations. Specifically, our objectives are to (1) identify and prioritize key design considerations for food recommenders that balance personalization, nutritional guidance, and user experience; and (2) conduct a proof-of-principle study in a real-life setting to assess the system’s effect on user understanding, trust, and potentially on dietary choices.

**Methods:**

An iterative, user-centered design process guided the development and refinement of the system across 4 phases: (Phase 0) an exploratory qualitative study (N=26) to understand stakeholder needs and initial system impressions, (Phases 1 and 2) rapid prototyping in real-life deployments (N=45 and N=16, respectively) to iteratively improve usability and features, and (Phase 3) a proof-of-principle study with employees (N=136) to evaluate a set of design goals. We collected a mix of data, including usage logs, pre- and post-study questionnaires, in-app feedback, and a pre- and post–Food Frequency Questionnaire to establish nutritional profiles.

**Results:**

Although we experienced a high drop-out (77% after 7 weeks), motivated and remaining participants valued personalization features, particularly the ability to configure allergies and lifestyle preferences. Explanations increased understanding of recommendations and created a sense of control, even when preferences and healthy options did not fully align. However, a mismatch persisted between individual preferences and nutritionally optimal recommendations. This highlights the design challenge of balancing user control, meal variety, and the promotion of healthy eating.

**Conclusions:**

Integrating explanations into personalized food recommender systems might be promising for supporting healthier food choices and creating a more informed understanding of dietary patterns. Our findings could highlight the importance of balancing user control with both the practical limitations of food service settings and the need for nutritionally sound recommendations. While fully resolving the tension between immediate preferences and long-term health goals is an ongoing challenge, explanations can play a crucial role in promoting more conscious decision-making.

## Introduction

Despite growing awareness of healthy eating, the prevalence of obesity tripled between 1975 and 2016 [1]. This disconnect, driven largely by unhealthy diets and malnutrition [2], suggests that traditional information campaigns often fail to translate knowledge into sustained behavioral change [3]. Since workplace food choices occur in controlled retail environments like catering and restaurants, foodservice providers have a unique opportunity to guide healthier decisions. However, current offerings in these settings often prioritize convenience over health. Integrating personalized dietary guidance with foodservice providers could be a significant step in promoting healthier eating habits within [4] and beyond the workplace [5].

Recent research emphasizes the need for personalized approaches to promote healthy eating [6-8]. Food recommender systems offer the potential to both empower people to monitor and improve their food-intake through technology-assisted, personalized healthy recommendations [9,10]. These systems can personalize suggestions, engage users to change their consumption behavior [11], and provide actionable insights based on individual eating patterns [12,13]. While studies indicate greater adherence to personalized dietary advice compared to generic guidelines [14], many people still face challenges in achieving their health goals.

Food recommender systems are software applications that leverage user data and algorithms to provide personalized suggestions for meals, recipes, or food items. Studies typically focus on suggesting recipes [15,16], meal plans [17], menu items [18], and more recently personalized nutrition [19]. However, as indicated by Trattner and Elsweiler [20], food recommender systems are relatively under researched compared to other recommender domains. There are many reasons that make food recommendations challenging [20,21], not only in terms of encouraging healthy behavior, but also in predicting what people would like to eat. The challenges are multifaceted, culturally determined, and context dependent. Users may have complex, constrained needs, such as allergies or life-style preferences, such as the desire to eat only vegan or gluten-free food. In such cases, standard recommender techniques often fall short in these contexts [22,23]. Data sources in real-life settings are often limited, for example, food catering services usually have restricted menu options. Other challenges include retrieving and selecting user-nutrition profiles, labor intensive food tracking, missing ingredients, and atypical for recommender systems, the most popular meals are not always the healthiest options [24]. For example, El Majjodi et al [25] found that commonly used, preference-based and popularity-driven approach could lead to a decrease in the healthiness of chosen recipes. To address these challenges, explanations within food recommender systems are crucial. Such explanations would clarify why certain recommendations are made, considering the user’s unique profile and health objectives.

Explanation interfaces have demonstrated positive impacts on user acceptance and trust in recommender systems [26,27]. A growing body of research highlights the benefits of explanations in enabling transparency and informed decision-making [28-30]. This focus on explanations is increasingly being applied in high-risk domains, such as health care [31] and job search [32], to support users in navigating complex recommendations. However, as recently highlighted by Musto et al [24], the use of personalized explanations in real-world food recommender systems remains underexplored. While some systems offer healthier alternatives [33,34] or incorporate user preferences [35], considerations around interface design and their impact on user choices are crucial [36]. In addition, few food recommender systems comprehensively integrate nutritional profiles [37], especially in real-life settings.

Although we experienced a high drop-out of 77% after 7 weeks, this paper makes an initial contribution by longitudinally deploying and evaluating an explanation interface within a food catering application. Research on user acceptance and trust of explanations in the food domain is limited [21], highlighting the need for studies that explore their potential to promote healthier eating habits. While early work [38] linked explanations to user preferences, or mainly evaluated system usability [4], our approach advances the field by tailoring explanations to individual nutritional profiles. Unlike studies requiring manual food tracking [39], our approach integrates explanations directly into the recommendation process.

This study aims to investigate the design, implementation, and evaluation of a food recommender system that integrates explanations in a real-world workplace setting. Our goal is to gain a deeper understanding of the user experience with explanations, specifically how they influence comprehension, trust, and ultimately, dietary decision-making. In addition, we seek to identify key design challenges and opportunities for balancing personalization with the promotion of healthy eating in the complex context of a real-life food service environment.

## Methods

### Study Design

**Figure 1.**
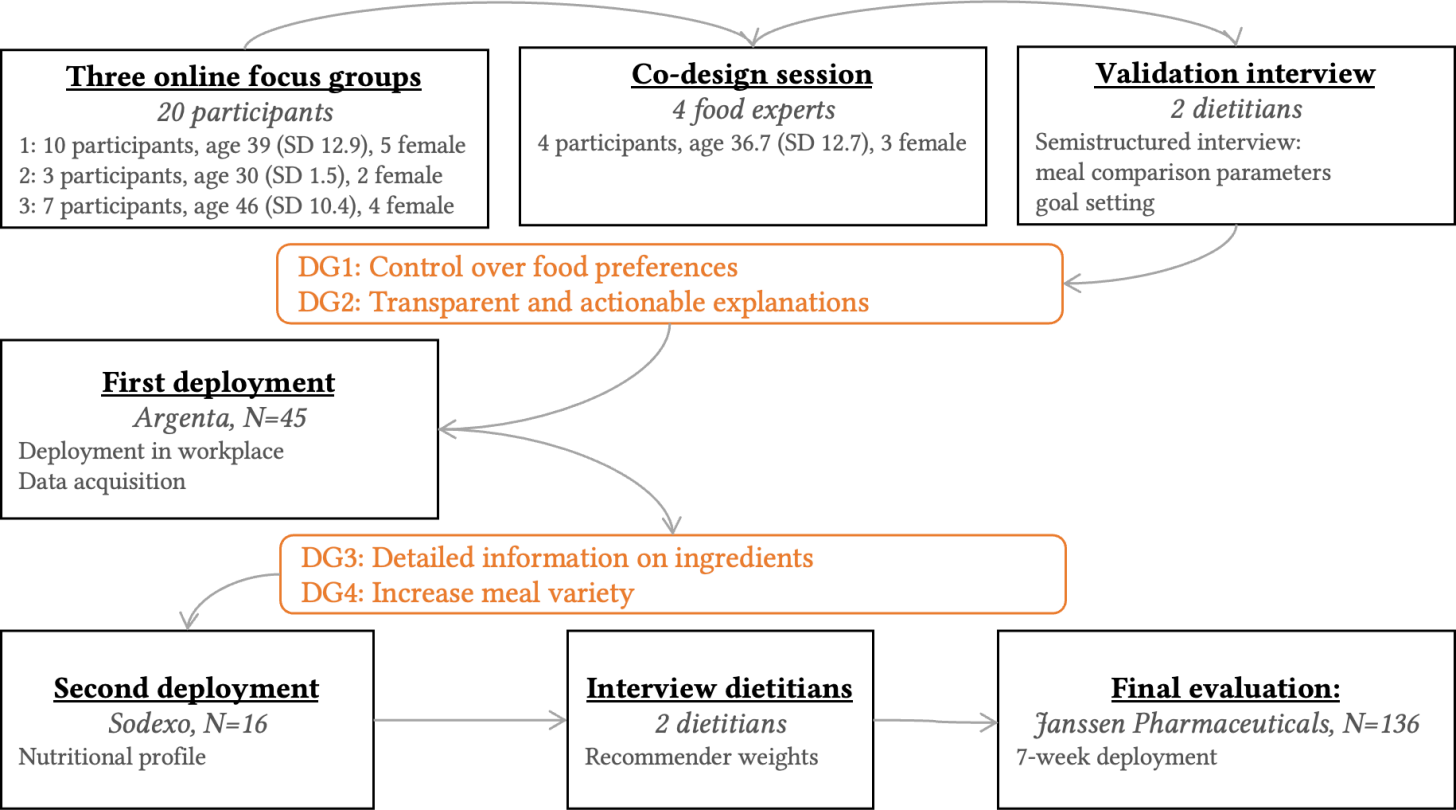
Timeline that shows the different steps. DG: design goals.

**Figure 2.**
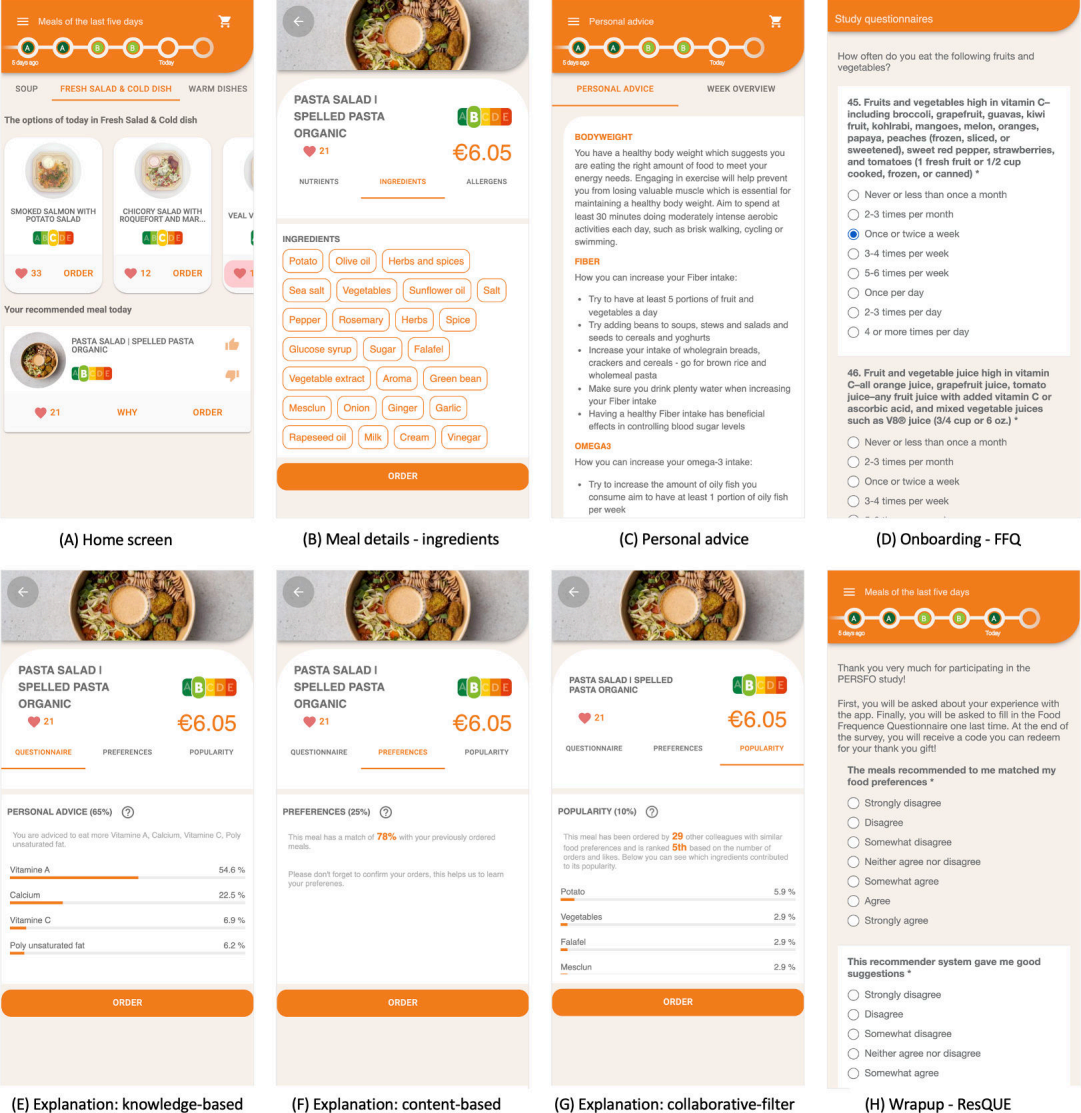
Screens and user interface components from the mobile application: (**A**) home screen shown after a user is logged in, (**B**) meal details shown when a user selects a meal, (**C**) personal advice based on the user’s nutritional profile, part of the (**D**) Food Frequency Questionnaire (FFQ), (**E**) knowledge-based explanation, (**F**) content-based explanations, (**G**) collaborative-filter explanations, and part of the final (**H**) ResQue questionnaire.

This study used an iterative (see Figure 1), user-centered methodology to design, implement, and evaluate a food recommender system with an integrated explanation interface (see Figure 2). We gathered feedback through a multiphase approach:

#### Phase 0: Initial Exploration and Requirement Gathering

To understand potential barriers to integrating a transparent food recommender system within a food catering application, we conducted a multiphase stakeholder analysis. Two lead researchers, in close collaboration with food and technical experts, designed a set of 25 user interface designs. These were evaluated in 3 focus groups (N=20), a co-design session (N=4), and a semistructured interview with 2 dietitians. Participants rated and discussed the designs. The complete results are described in [40].

#### Phase 1 and 2: Data Acquisition and Rapid Prototyping

The goal of Phases 1 and 2 was to develop, deploy, and refine the system in real-life settings, gathering feedback on usability and functionality. The system was iteratively deployed at 2 companies chosen in collaboration with our food service partner (to ensure access to the necessary food management systems)—a bank (Phase 1, Argenta, N=45) and a facilities management company (Phase 2, Sodexo, N=16). These deployments with a diverse participant pool informed refinements of both the interface and the recommender engine before the proof-of-principle study.

To address the common “cold start” problem (ie, not enough initial data to create a user profile) in health recommenders [41] and personalize recommendations, we integrated a food frequency questionnaire (FFQ) [42]. FFQs offer a more comprehensive view of dietary habits than short-duration food records [43] and have demonstrated validity in previous research [44]. We used a validated FFQ [45-47] and the European Union–funded Quisper platform [48] to generate personalized nutritional profiles [49,50]. The Quisper Food4Me service provided personalized feedback (Low, Normal, or High nutrient intake) based on pseudoanonymized FFQ data. Interaction logs and in-app user feedback were collected to inform system improvements. Ethical approval was granted by the university’s ethics committee (approval number G2019-12-1911).

#### Phase 3: Proof-of-Principle Study

The goal of this third and final phase was to evaluate the system’s impact on user understanding, trust, and dietary choices in a larger deployment. The refined system was deployed in a large pharmaceutical company restaurant (Janssen Pharmaceuticals, N=136). Pre- and post-study FFQ, the ResQue questionnaire, interaction logs, and open feedback functionality were used to collect comprehensive data about system usage, user perceptions, and potential dietary changes. Ethical approval was granted by the university’s ethics committee (approval number G2021-3055-R2(MIN)).

### Mobile Application and Explanation Interface

The home screen (Figure 2A) provides an overview of daily menu options, including courses (eg, soup, salads, etc), individual meals, and their nutriscore [51], a color-coded nutritional label that indicates the overall healthiness of a food item. Scores range from green (A, most nutritious) to red (E, least nutritious), helping users make informed choices at a glance. Users can tap a meal for detailed nutritional information, ingredients (Figure 2B), and allergens. Personalized dietary advice is accessible through the “Personal advice” screen (Figure 2C).

Users express preferences with a thumbs-up icon for liked recommendations (Figure 2A). If they disliked a recommendation, they were asked which ingredients contributed to their dislike. The why button reveals the following 3 explanations:

1. Nutritional alignment (Figure 2E): This explains how the recommended meal aligns with the user’s nutritional profile and the weighting of each nutrient. A question mark icon offers additional context (eg, “Your recommendations are 65% influenced by your nutritional profile.”).
2. Past choices (Figure 2F): This highlights how the recommendation relates to the user’s previous orders and liked meals. A question mark icon clarifies the process (eg, “Based on your previously chosen meals, we try to find similar recipes.”).
3. Popularity (Figure 2G): This reveals the meal’s popularity among similar users and emphasizes that popularity is a minor recommendation factor (eg, “The most popular meals are not always the healthiest meals. Therefore, the popularity of a meal is only taken into account for 10%.”).

### Hybrid Recommender Engine

**Figure 3.**
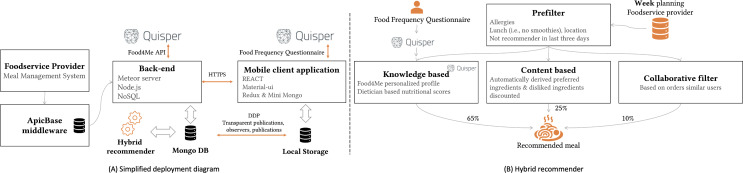
Schematic overview of the (A) deployment diagram and (B) the different hybrid recommender building blocks.

Our food recommender system (Figure 3A) integrates multiple strategies to personalize meal suggestions in real-world foodservice settings, with a focus on promoting healthy choices. Weekly menus designed by the main chefs of the company restaurants were accessed through standardized interfaces (RESTful API) and stored in a flexible database system (MongoDB). The underlying hybrid food recommender engine (Figure 3B) consists of 4 key building blocks:

#### Contextual Prefilter

The menu is initially filtered to ensure relevance and safety, considering:

- Location-specific offerings.
- User-reported allergies, promoting dietary safety.
- Recent recommendations (previous 3 days) to enhance meal variety and prevent repetition.

#### A Knowledge-Based Recommender

Leveraging the Quisper Food4Me integration [49], we generate personalized nutritional profiles that guide meal rankings. Users receive personalized feedback (Figure 2D and 2E), promoting dietary awareness. Based on the input from the stakeholder analysis, this emphasis on nutritional alignment accounts for 65% of the final recommendation score.

#### Content-Based Engine

A cosine-similarity algorithm assigned a personalized score to each meal based on the historical purchases of the user. Using the thumb-up and down icons, users were provided with additional controls to impact their recommendations. This content-based engine thus mainly assisted in differentiating between preferred options. The ranking score, therefore, only contributed 25% to the final ranking score.

#### Collaborative-Filter

After 2 weeks of data collection, a nearest-neighbor algorithm suggests meals based on the order histories of users with similar dietary patterns. Due to the intervention period, this collaborative element contributes 10% to the final recommendation score.

### Data Analysis and Recruitment

Qualitative data derived from the focus groups, co-design session, semistructured interviews, and in-app user feedback was analyzed using thematic analysis and the results are described in [40]. This analysis focused on identifying key themes related to user needs, preferences, design considerations, usability issues, and overall system perceptions. Interaction log data from all system deployments was analyzed using descriptive statistics. This analysis examined user engagement patterns (frequency of use, feature popularity, and interaction with meal recommendations) and provided insights into user behavior within the system. In Phase 3, data from the ResQue questionnaire [52] was analyzed using descriptive statistics to assess user perceptions of the explanations, their sense of control, and the system’s impact on trust. Dietary intake data obtained through the FFQ at baseline and after the intervention was analyzed to examine potential changes in food choices and nutrient intake. We analyzed this data using the Belgian Food Composition Database (NUBEL) [53]. This analysis focused on comparing food group consumption and shifts in macronutrient proportions. User comments from the in-app feedback feature were analyzed to gain additional insights into user experiences, preferences, and suggestions for improvement.

### Recruitment Strategies

Phases 1 and 2 prioritized usability testing and formative evaluation of the recommender system interface. Here, our primary goal was to gather feedback from a diverse range of users within each organization. This user diversity was essential for identifying usability issues, refining the interface, and optimizing the recommender engine’s functionality. In this context, a group interested in exploring the system’s features was more valuable than a perfectly representative sample of the overall employee population. For the later, proof-of-principle study (phase 3), we adopted a broader recruitment approach (emails likely supplemented by existing communication channels within the organizations) to ensure a larger, and more representative participant pool. While self-selection remains a limitation (more details in the Limitations section), our main objectives were to (1) identify key design considerations for balancing personalization, nutritional guidance, and user experience in food recommenders; and (2) assess the system’s impact on understanding, trust, and dietary choices in a real-life setting.

### Ethical Considerations

Participation was voluntary and fully anonymous in all phases to protect sensitive health data. The restaurant proprietor managed recruitment and communication with participants, while we handled only anonymized data, ie, participants were asked to use a pseudonym when creating an account on the study platform. Informed consent was obtained, emphasizing participant anonymity and secure data handling. Personally identifiable information was delinked from usage and dietary data as early as possible, and data was stored on secure servers with access limited to authorized researchers. The university ethics committee reviewed and approved all studies. Ethical approval for phase 1 and 2 was granted by the university’s ethics committee (approval number G2019-12-1911). Ethical approval for phase 3 was granted by the university’s ethics committee (approval number G2021-3055-R2(MIN)).

## Results

### Phase 0: Initial Exploration and Requirement Gathering

The complete results of the user centered design studies in Phase 0 are described in [40] and they provided insights into the needs of various stakeholders. This analysis yielded 2 key design goals (DG):

#### DG1: Control Over Food Preferences

End users and other stakeholders desire a food experience that balances authenticity, convenience, health, and environmental consciousness. The platform should accommodate varied preferences, including:

- Lifestyle and environmental: options such as vegan, halal, locally sourced food.
- Medical: clear allergen information.

#### DG2: Transparent and Actionable Explanations

Recommendations and explanations should enhance informed decision-making. Users expect control over food choices, requiring:

- Transparency: Clear reasoning behind recommendations.
- Actionable insights: Guidance on how recommendations align with individual preferences and health goals.

### Phase 1: Data Acquisition

To test technical integration and gather data to train a recommender algorithm, we deployed an online, but fully working, mobile prototype (adapted due to COVID-19 restrictions) at Argenta Headquarters Belgium (December 7‐18, 2020). Collaborating with the head chef, we designed a menu mirroring restaurant offerings. During the 2-week study, 45 participants placed 427, with an average of 9 orders per user and 9066 logged interactions. This yielded valuable technical insights and user feedback, leading to 2 additional design goals.

#### DG3: Fine-Grained Ingredient Data

Technical integration revealed limitations in the available ingredient data. Ingredient lists were often composite (eg, Bolognese sauce, without specifying its constituent ingredients) and precise portion sizes were unknown. This lack of granular data hinders effective ingredient-based recommendations [54]. In total. 10 participants echoed this concern, expressing difficulty making informed choices due to missing ingredient details: “I think it would be useful to list all ingredients in the description. For example, I see in the picture that there are pomegranate seeds with the salad, but that is nowhere to be read. Maybe it contains ingredients that someone is allergic to.” Another participant highlighted the need for low-level ingredients: “Pasta with Alfredo sauce: how the hell do I know what Alfredo sauce is? The photo or the nutritional values give me little or no info about that. I want to know what that is right (a kind of cheese sauce?) and what the predominant taste will be.”

#### DG4: Increase Meal Variety

Although the menu used was prepared by the head chef as it would have been served in the restaurant, participants voiced concerns about limited healthy options, specifically a lack of vegetarian, gluten-free, and vegan choices. Comments like, “also today I find the healthy range too limited, far too little focus on vegetables;” or “it strikes me that there is little choice for people who really want to eat healthy, for example, there are virtually no vegetarian / gluten-free dishes or vegan options,” highlight the need for greater meal variety to support diverse dietary needs.

### Phase 2: Rapid Prototyping and Quisper Integration

We deployed an enhanced prototype for one week at Sodexo headquarters (December 3‐10, 2021). A total of 16 participants completed the FFQ and received personalized feedback through the Food4Me Quisper service [49] that was tailored to local food. Users provided feedback on recommendations thumbs up and down icons or open feedback field. The main goal was to collect data for refining personalized recommendations based on nutritional profiles.

Our findings highlighted the need to balance personalized nutritional advice with the actual nutrient content of meals. We normalized meal nutritional values and developed a weighted scoring system with input from 2 professional dietitians (see Figure 1, Table 1). This aimed to optimize recommendations based on individual needs. For example, a person with low fiber intake would receive a higher score fiber-rich meals (weight=5). However, when fiber intake was already high, fiber-rich meals would not be further emphasized (weight=0). In contrast, when a person does not consume a lot of polyunsaturated fats, meals with a higher amount of polyunsaturated fat could receive a slightly higher ranking (weight=3). When a person consumes enough polyunsaturated fats, meals with a higher amount should be discounted (weight=−0.5). This approach, detailed in Table 1, ensures that recommendations align with individual user profiles without neglecting important dietary considerations.

**Table 1.** Nutrient weights for personalized recommendations. Higher weights for “low intake” indicate nutrients the user needs more of. Negative weights for “high intake” indicate nutrients the user should consume in smaller amounts.

Nutrient	Low intake, weight	High intake, weight
Fiber	5	0
Vitamin A	2	0
Vitamin B12	3	0
Vitamin C	5	0
Monounsaturated fat	1	0
Polyunsaturated fat	3	−0.5
Proteins	3	−0.3
Total fat	−1	−1
Calcium	5	0
Iron	2	0
Saturated fats	0	−1
Carbohydrate	3	−0.5

### Phase 3: Proof-of-Principle Study

Our final proof-of-principle study ran from January 17, 2022 to March 4, 2022 at Janssen Pharmaceuticals. Each participant was asked to use the platform for 7 weeks and order their meals through the application, 176 employees initially expressed interest and 136 (77%) participants filled in the full FFQ and made at least 2 orders through the study application. Participants ranged in age from 23 to 61 (average age 41, SD 10 y), with 65% (88/136) female participants. Their BMI and weight followed a normal distribution. Despite COVID-19 restrictions limiting on-site work, participants made a mix of real and simulated food orders. We logged 17,597 interactions and 1031 orders placed by the app, demonstrating active usage. After completing the study, 41 participants who participated for 7 weeks and filled in the final questionnaires received a $42 (€40) healthy snack box and personalized dietary advice, similar to Figure 2C.

#### DG1: Control Over Food Preferences

User interaction logs demonstrate that users actively consulted and used the option to configure their own food preferences. Results show that participants consulted personalized recommendations and tried to keep their profile up to date: “I sometimes skip lunch due to time restricted feeding. There is no option to save this in the application.” The most requested feature was to log meals from previous days or to add own options: For example, “you cannot enter fruit or yogurt into the system, which can also be useful. If necessary, you provide something that you can type yourself.”

The food service provider supported 34 allergies (see Textbox 1) and 31 lifestyle preferences, yet some participants indicated they would have preferred even more options: “My allergy is not in the list. Tomatoes and all preparations with tomato (such as with cocktail sauce, tomato powder in eg, marinades.)” Other potential features were options to integrate with existing diet programs, such as Weight Watchers, or the option to differentiate between a “cold” and “warm” dish: “I also like to eat warm, and I was always advised a cold dish [=salad].”

Textbox 1. **Food preferences supported by the platform.**

- Allergy: Barley, fish, lupine, mollusk, corn, wheat, sulfides, gluten, Brazil nuts, shellfish, mustard, sunflower seeds, egg, hazelnuts, celery, spelt, pistachio nuts, macadamia nuts, nut, lactose, poppy seeds, pecan nuts, rye, milk dairy, sesame, oats, walnuts, peanut, legume pulse, Khorasan wheat, cashews, soy, almonds, and seeds.

- Preference: Coriander, taurine, coloring, sulfur, phosphate, caffeine, nitrates, sorbates, alcohol, poultry meat, blackened, halal, beef, benzoates, kosher, acesulfame e962, vegan, aspartame e951, lamb, sweetener, preservative, pig meat, antioxidant, genetic modified, flavor enhancer, glutamate, quinine, vegetarian, carrot, cacao, and propionates.

High-level dietary intake analysis of the 41 motivated participants who finished the 7-week study revealed positive initial trends in food choices. After the intervention, we observed an increased contribution of vegetables and fruits to total carbohydrate intake in several participants (3 and 1, respectively). This was accompanied by a decrease in reliance on starchy carbohydrates like potatoes, rice and pasta (6 at baseline, 3 after) and bread or savory biscuits (1 at baseline, 0 after). Protein intake patterns also shifted, with decreased reliance on dairy (3 at baseline, 2 after) and sweets or snacks (1 at baseline, 0 after), alongside increased vegetable intake (2 at baseline, 4 after). Differences in bread and savory biscuits (1 at baseline, 0 after); dairy (1 at baseline, 2 after); fats and spreads (7 at baseline, 10 after); meat and fish (17 at baseline, 13 after); and potatoes, rice, and pasta (0 at baseline, 1 after) were found when estimating food group contributions toward total fat intake.

#### DG2: Transparent and Actionable Explanations

**Figure 4.**
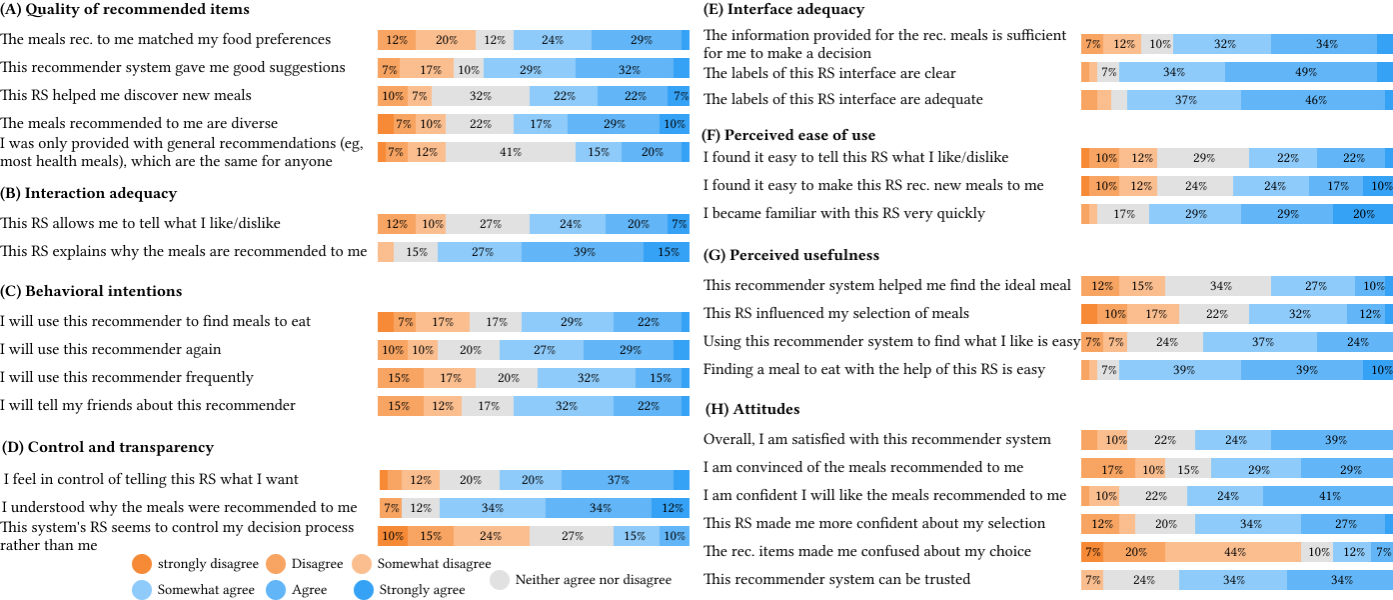
Results from the final questionnaire: (**A**) quality of recommended items, (**B**) interaction adequacy, (**C**) behavioral intentions, (**D**) control and transparency, (**E**) interface adequacy, (**F**) perceived ease of use, (**G**) perceived usefulness, and (**H**) attitudes. RS: recommender system.

ResQue questionnaire results (Figure 4, N=41) revealed a mismatch between what people wanted to eat and their healthy recommendations. Most respondents (33/41) found the explanations clear (36/41) and adequate (33/41) in explaining why meals were suggested. This understanding extended to the food recommender system and, 32/41 understood why meals were recommended to them, 32/41 indicated they quickly became familiar with the mobile user interface and found it easy to find a meal with the help of the app (36/41). Thanks to the explanations (Figure 2E), users received visual insight into their own dietary patterns and gained insights how the recommendations aligned with their nutritional needs.

However, the mismatch remains evident: only 22/41 felt the recommendations matched their food preferences. Despite this, explanations seem to have mitigated potential negative impacts.

#### DG3: Fine-Grained Ingredient Data

Despite its importance to users, many food service providers lack detailed ingredient data. This study confirmed the need for ingredient breakdowns and precise portion information. Interaction logs revealed frequent use of the ingredient feature (961 views, 83% of orders), yet participants expressed a desire for more granularity. For example, one participant requested a breakdown of sugar types (glucose, fructose, etc) to support their diet. Interestingly, 69% (28/41) respondents found the provided information, including the summary information of the nutriscore [51], sufficient for making informed decision. Positive feedback like, “Definitely a nice system!” highlights the nutriscore’s potential in aiding quick assessment of a meal’s healthiness.

#### DG4: Meal Variety

Our real-life deployment of 7 weeks highlighted a challenge in balancing meal variety with personalized recommendations. The restaurant expanded its menu options during the study, but daily offerings remained limited (10‐20 meals). Consequently, only half of participants perceived the recommendations as diverse. Most did not feel the system promoted generic options (14/41), and only 21/41 found it helpful for discovering new dishes. However, the extensive allergy and lifestyle filters further reduced recommendation variety for some users, leading to a perception of repetitiveness (eg, “It was unfortunate that I almost always got the same advice.”). This reflects the design’s focus on addressing individual nutritional needs. For example, when a participant scored low or high on a certain nutritional intake, they were recommended meals that contained more or less of that nutrient. After all, nutritional uptake is not “fixed” with 1 meal. This was also experienced by the participants: “I find it strange that I always get similar suggestions, there has never been a dish of the day or a salad of the week recommended [...]. This is very monotonous isn’t it?” Despite this perceived monotony, 58% (24/41) of motivated participants liked the meals recommended to them and 65% (27/41) expressed confidence in future recommendations.

## Discussion

### Principal Findings

While the initial interest in the study was high (176 employees), the actual engagement was lower, with 136 participants (77%) completing the FFQ and actively using the application. It is also important to highlight that of these 136 participants, only 41 completed the ResQue questionnaire and the final FFQ after 7 weeks. This attrition rate is consistent with observations in other digital health interventions, which often face challenges in maintaining user engagement over time. This further reduction in participants was likely due to the extended length of the study and the requirement to consistently use the application for ordering meals. Therefore, it is important to note that the results from the ResQue questionnaire and the observed dietary changes are based on this subset of 41 participants. Despite the lower-than-expected uptake, the study provided valuable lessons learned into user preferences, design considerations, and the potential of personalized explanations to promote healthier food choices. Future research with a longer intervention period and post–follow-up is needed to fully assess sustained behavioral change and address the engagement challenges.

### Lessons Learned

Our preliminary study suggest that motivated users might value user control features (DG1), such as allergy filters, feedback mechanisms, and personalized nutritional profiles. However, participants desired even greater customization, including additional allergy options, integration with dietary programs, and finer control over meal types (eg, warm or cold, only a sandwich during lunchtime). While increased control could potentially enhance meal variety (DG4), it must be balanced against practical limitations; for example, too strict food preferences limited the available meal options (eg, only 1 or 2 vegan options per day), and the potential for users to inadvertently undermine their own nutritional goals. Implementing meal customization options could potentially further increase meal variety, for example, “the choice of the type of sandwich would make up for a lot.” But this has a lot of pragmatic implications, such as pricing and supply chain issues. Customization would also impact the nutritional values and require cumbersome actions from users to track a correct nutrition intake profile. Furthermore, increasing meal variety is more complex than simply introducing more variety in the menu options, dietary changes require an increased or decreased intake of the same user specific nutrients over a longer period of time [39].

The automatically generated nutritional profiles were crucial for the acceptance and trust (DG2). Motivated participants actively consulted profiles, tried to understand recommendations, and performed explicit actions to keep their nutritional profile up to date (eg, they even contacted the research team when they forgot to register a meal). However, at least 27% (11/41) felt the recommendations did not help them find healthier options, which could be due in part to their allergy or other lifestyle related filters, and 37% (15/41) felt recommendations were too general. We managed to support 80% (33/41) of remaining participants to understand their healthy food recommendations. However, consistent with previous research [29,47], we observed a mismatch between what people liked to eat and their healthy recommendations. While personalized nutritional profiles and explanations could have the potential to increase awareness of healthy eating, they cannot guarantee that all users will consistently opt for healthier choices.

### Healthy Food Recommendations? Thanks, but No Thanks - Design Implications

Our study presents preliminary insights into the crucial dilemma between perceived user control and recommendation diversity, impacting both meal variety and potential dietary impact. Self-determination theory [55] underscores the importance of autonomy for lasting behavior change. The more individuals feel they understand their own eating habits, the more likely they are to adopt a healthy diet and achieve physical and psychological health [56]. However, if food recommenders mainly take user preferences into account, bad eating habits could be encouraged [21]. Furthermore, as our results demonstrate, excessive focus on preferences can reduce meal options, limiting variety and potentially hindering dietary change.

Prioritizing either nutrients or meal variety has implications for the system’s effectiveness. Dietary changes can only be achieved after there is a significant increase (eg, calcium) or decrease (eg, saturated fats) of certain nutrients. However, emphasizing nutrient optimization could lead to recommendations users find unpalatable or repetitive, reducing perceived control [21,57]. Conversely, focusing solely on variety risks reinforcing existing preferences, even unhealthy ones [21].

By helping users understand the rationale behind recommendations and their connection to individual nutritional profiles, we could potentially increase perceived control and potentially motivate change. Therefore, a successful food recommender design must carefully balance control features with personalized nutritional guidance [21]. Clear explanations can support understanding and could create a sense of autonomy, increasing acceptance and trust in the system.

### Limitations and Future Work

There are important limitations to this preliminary work that need to be acknowledged.

First, participants were self-selected for greater interest in healthy eating, introducing bias. Despite this, our design implications remain valuable for supporting motivated users, who are the most likely adopters of food recommender applications. Our focus on human-computer interaction methods aims to address the gap between healthy eating motivation and action, making our findings relevant to this target audience.

Second, COVID-19 restrictions necessitated a mix of real and virtual orders. Virtual orders may reflect idealized choices rather than true consumption habits. However, we have examined if any significant trends exist between order types (simulated vs actual) related to both the healthiness of choices and user perceptions captured by the ResQue questionnaire. No difference could be observed within the group of 41 participants that filled in the final questionnaires. It is also worth noting that the abovementioned selection bias, could have positively influenced the similarity in trends between simulated and real orders. Participants who were genuinely interested in exploring healthier eating may have been more conscientious about both their real and virtual meal choices, minimizing the potential discrepancy. While this does not negate the limitations for translating virtual choices into real-world dietary change, it suggests a level of engagement that benefits the exploration of system design elements. Nonetheless, a longer intervention with post–follow-up is needed in future work to fully assess sustained behavioral change.

Finally, the field of transparent recommender systems is evolving [26], and comparative studies exploring alternative explanation methods, such as multilist interfaces [57], could offer insights into greater effectiveness. Future research possibilities include a more advanced content-based algorithm using detailed ingredient data (currently the exact ingredient contributions were not available for every meal); and the ability for users to log meals consumed outside the company restaurant for a more complete nutritional assessment.

## Abbreviations

DG: design goals
EIT: European Institute of Innovation and Technology
FFQ: Food Frequency Questionnaire
